# ETV1 transcriptional manipulation of KIFC1 regulates the progression of pancreatic cancer

**DOI:** 10.32604/or.2025.059631

**Published:** 2025-06-26

**Authors:** FANGFANG HU, ZHIBIN BAI, YANG WANG, HAODONG TANG, JIAHUA ZHOU

**Affiliations:** 1 Department of Hepatobiliary and Pancreatic Surgery, Zhongda Hospital, Medical School, Southeast University, Nanjing, 210009, China; 2 Center of Interventional Radiology and Vascular Surgery, Department of Radiology, Zhongda Hospital, Medical School, Southeast University, Nanjing, 210009, China; 3 Medical School, Southeast University, Nanjing, 210009, China

**Keywords:** ETV1, KIFC1, PI3K/AKT, Pancreatic cancer, Invasion and migration

## Abstract

**Background:**

Kinesin-14 family protein 1 (KIFC1) is abnormally overexpressed in various cancers, and the transcription factor ETS variant 1 (ETV1) is an oncogenic transcription factor in tumors. The potential binding sites on the KIFC1 promoter by ETV1 were observed; however, no evidence supports that ETV1 targets KIFC1. Aims: This study aimed to investigate the relationship between KIFC1 and ETV1, and their effects and mechanisms in pancreatic cancer.

**Methods:**

Pan-cancer analysis of KIFC1 expression was performed in GEPIA2 database. KIFC1 expression levels were determined by immunohistochemistry (IHC) in our pancreatic cancer cohort. The correlation between KIFC1 expression and prognosis, tumor mutation burden, tumor purity, mismatch repair, and high-frequency tumor mutated genes was analyzed using a series of bioinformatic tools. ETV1 targeting of KIFC1 promoter transcription was determined using luciferase reporter assay. KIFC1 knockdown and ETV1 overexpression were used to determine the role of the ETV1/KIFC1 axis in cell proliferation, migration, invasion, and epithelial-mesenchymal transition (EMT) of pancreatic cancer cells *in vitro* and tumor growth *in vivo*.

**Result:**

KIFC1 expression was increased in clinical specimens and pancreatic cancer cell lines and positively correlated with tumor mutation burden, tumor purity, mismatch repair, and KRAS and TP53 mutations. High KIFC1 expression was significantly associated with poor prognosis. Knockdown of KIFC1 suppressed the proliferation, migration, and invasion of pancreatic cancer cells and tumor growth. ETV1 overexpression increased KIFC1 expression and affected KIFC1 transcription. ETV1 overexpression reversed the role of KIFC1 knockdown in inhibiting cell proliferation, invasion, migration, and EMT, as validated *in vivo*.

**Conclusions:**

KIFC1 serves as a tumor activator in pancreatic cancer by promoting proliferation, migration, invasion, and tumor growth, which may be partly manipulated by ETV1.

## Introduction

Pancreatic cancer is a highly malignant and lethal tumor, killing more than 466,000 people worldwide each year and resulting in a 5 years survival rate of less than 10% [1,2]. Along with substantial progress in the understanding of its biology, the incidence and number of cancer-related deaths have declined; however, pancreatic cancer has a poor prognosis because it is not amenable to advanced resection and responds poorly to most chemotherapy drugs [3]. Understanding the biological mechanisms that contribute to the development and progression of pancreatic tumors will provide new hope for treating patients with pancreatic cancer.

The kinesin-14 family (KIFC) is a group of microtubule motor proteins with ATPase activity that play a role in cell division, cytokinesis, cell proliferation, and apoptosis [4,5]. KIFC1 is a membrane of the kinesin-14 family and is involved in processes of centrosome aggregation, microtubule transport, and spindle formation during mitosis [6,7]. Recent studies have suggested that KIFC1 is overexpressed in various cancers and that abnormally overexpressed KIFC1 plays a key role in promoting cancer progression. For example, KIFC1 is highly expressed in hepatocellular carcinoma and promotes hepatocellular carcinoma cell proliferation and invasion [8,9]. High KIFC1 expression in gastric cancer promotes proliferation, migration, and invasion of gastric cancer cells [10]. High expression of KIFC1 in ovarian cancer promotes proliferation, migration, and epithelial-mesenchymal transition (EMT) in ovarian cancer cells [11]. These studies highlight the role of KIFC1 in promoting cancer cell proliferation, invasion, and migration. However, the role of KIFC1 in pancreatic cancer remains largely unexplored.

Transcription factor ETS variant 1 (ETV1) has been recognized as an oncogenic transcription factor in a wide variety of tumors. Increasing number of studies reveal the crucial role of ETV1 in colorectal and prostate cancers [12–14]. A previous study observed increased ETV1 expression in human pancreatic cancer tissues and found that tissues with ETV1 overexpression underwent EMT and had enhanced invasive capacity [15]. Considering the mechanism, the role of ETV1 in tumorigenesis attributes to its promoting function on target cancer-promoting gene promoter transcription [12,15]. Although no evidence supports ETV1 targeting of KIFC1, we observed potential binding sites on the KIFC1 promoter for ETV1.

Therefore, the current study clarified the expression pattern of KIFC1 in pancreatic cancer tissue specimens and cell lines and explored its role in the proliferation, invasion, and migration of pancreatic cancer cells, as well as tumor growth *in vivo*. Further experiments focused on the transcriptional manipulation of ETV1 in KIFC1 cells. These results suggest a potential therapeutic target for pancreatic cancer.

## Materials and Methods

### Pancancer analysis of KIFC1

The expression levels of KIFC1 in different cancer types and corresponding controls in The Cancer Genome Atlas Program (TCGA) and genotype-tissue expression (GTEx) were determined using the Gene Expression Profiling Interactive Analysis (GEPIA) database (http://gepia.cancer-pku.cn/, accessed on 5 March 2025).

### Correlation of KIFC1 with cinical factors of pancreatic cancer

Pancreatic cancer samples in TCGA were divided into high- and low-expression groups according to the median expression value of KIFC1. Statistical differences in clinical factors between the high- and low-expression groups were calculated using Fisher’s exact test in R4.1.2. The association between KIFC1 expression and prognosis was analyzed using the Kaplan–Meier method in the survival package (http://bioconductor.org/packages/survivalr/ (accessed on 5 Mar 2025), version 2.41-1) of R4.1.2. The diagnostic value of KIFC1 was further evaluated using the receiver operating characteristic (ROC) curve in pROC (https://cran.r-project.org/web/packages/pROC/index.html (accessed on 5 March 2025), version 1.12.1) in R4.1.2. Univariate and multivariate Cox regression analyses were conducted using the survival package (http://bioconductor.org/packages/survivalr/ (accessed on 5 March 2025), version 2.41-1) in R4.1.2, to identify independent prognostic factors for pancreatic cancer.

### Correlation of KIFC1 with tumor mutation burden (TMB) and tumor purity

Gene mutation information from the TCGA database was downloaded, and the TMB of pancreatic cancer samples was calculated using the maftools package (https://bioconductor.org/packages/release/bioc/html/maftools.html (accessed on 5 March 2025), Version 2.6.05) [16] in R4.1.2. The correlation between the expression level of KIFC1 and TMB of the samples was calculated using the Cor function. Tumor purity was evaluated using the estimate package (https://sourceforge.net/projects/estimateproject/) (accessed on 5 March 2025), and the correlation between the expression level of KIFC1 and tumor purity was calculated.

### Correlation of KIFC1 with mismatch repair (MMR)

MMR genes are a class of tumor-related genes involved in mismatch repair response in humans [17]. The correlation between KIFC1 expression and a few well-established MMR genes in pancreatic cancer, including *MLH1*, *MSH2*, *MSH6*, *PMS2* and *EPCAM* was calculated using the Pearson correlation coefficient (PCC) method, and a correlation heatmap was generated.

### Correlation analysis of KIFC1 with tumor high frequency mutated genes

The genes of *KRAS*, *TP53*, *CDKN2*, and *SMAD4* are mutated in several pancreatic cancers. To further investigate the correlation between these gene mutations and KIFC1 expression, the mutation information of pancreatic cancer was downloaded from TCGA database (https://portal.gdc.cancer.gov/repository) (accessed on 5 March 2025). Then, the maftools version 2.6.05 (https://bioconductor.org/packages/release/bioc/html/maftools.html) (accessed on 5 March 2025) in R4.3.1 was employed to analyze the mutations of *KRAS*, *TP53*, *CDKN2*, *SMAD4*, and *KIFC1*, and visualize the gene locations.

### Gene set enrichment (GSEA) analysis of KIFC1 associated kyoto encyclopedia of genes and genomes (KEGG) pathways

Based on whole-genome expression data of pancreatic cancer samples from TCGA, the KEGG signaling pathways significantly associated with KIFC1 expression were screened using GSEA (http://software.broadinstitute.org/gsea/index.jsp) (accessed on 5 March 2025) [18]. In GSEA, three key statistical values are present: enrichment score (ES), normalized enrichment score (NES), and nominal *p*-value. In general, the greater the absolute value of NES and the smaller the nominal *p*-value, the higher the enrichment of gene sets, and the higher the reliability of the results. The significantly enriched KEGG pathways with false discovery rate (FDR) values less than 0.05 were finally selected.

### Clinical cancer tissues

Pancreatic cancer tissues were obtained from 20 patients who underwent surgical tumor resection at our hospital, with 20 paired para-carcinoma tissues as controls. Samples were obtained after obtaining patient consent. The specimen acquisition and protocols were approved by the Institutional Ethics Committee of Zhongda Hospital, Affiliated to Southeast University (approval no. 2018ZDSYLL142-P01).

### Immunohistochemistry (IHC)

Tissues were embedded in paraffin and sectioned. Sections were sequentially immersed in a series of xylene and ethanol solutions for dewaxing. After rehydration with a series of concentrations of ethanol solution (95%, 95%, 80%, 70%, and double distilled water), antigen repair was performed using a boiled sodium citrate solution for 10 min. Endogenous enzyme activity was inactivated by treatment with hydrogen peroxide. A blocking solution (goat serum, cat. no. abs933, Abisin, Shanghai, China) was dropped onto the sections to block nonspecific binding sites. The primary antibodies against KIFC1 (20790-1-AP, Proteintech, Wuhan, China), E-cadherin (20874-1-AP, Proteintech), Vimentin (60330-1-Ig, Proteintech), N-cadherin (BM1573, Boster, Wuhan, China), and Ki67 (27309-1-AP, Proteintech) were diluted at a 1:100 ratio and used to incubate with the section at 4°C overnight. The next day, the sections were incubated with secondary antibody (1:5000, cat. no. 111-035-003, or cat. no. 115-035-003, Jackson ImmunoResearch, Lancaster, PA, USA) at room temperature for 10 min. The targeted proteins in the sections were stained with diaminobenzidine (DAB) solution (cat. no. D7370, Solarbio, Beijing, China) and counterstained with hematoxylin (cat. no. H8070, Solarbio). The stained sections were dehydrated using a series of alcohol and xylene solutions and sealed with neutral resin. Finally, images were acquired using an optical microscope (model: IX70, Olympus Corporation, Tokyo, Japan). The section positive grade score was calculated as positive cell score × intensity of positive staining. Scores of 0, 1–4, 5–8, and 9–12 represented negative, weakly positive, positive, and strongly positive, respectively.

### Cell lines

The human normal pancreatic ductal cell line HPDE6-C7 and three pancreatic cancer cell lines, PANC-1, BXPC-3, and ASPC-1, were obtained from the Cell Bank of the Chinese Academy of Sciences (Shanghai, China). Based on the verification test, the cells did not contain mycoplasma, and based on Short Tandem Repeat (STR) results, the cell lines were indeed the HPDE6-C7, PANC-1, BXPC-3, and ASPC-1 cell lines. HPDE6-C7 and PANC-1 cells were cultured in Dulbecco’s Modified Eagle’s medium (DMEM; cat. no. 12491015, Thermo Fisher Scientific, Waltham, MA, USA) whereas BXPC-3 and ASPC-1 cells were cultured in RPMI1640 (cat. no. 12633020, Thermo Fisher Scientific). Both media were supplemented with 10% fetal bovine serum (FBS, cat. no. 16000-044, Thermo Fisher Scientific) and 1% penicillin and streptomycin (cat. no. 15140-122, Thermo Fisher Scientific). The culture conditions were set at 37°C and 5% CO_2_.

### Constriction of KIFC1 knockdown and overexpression by cell transfection

KIFC1 was knocked down by small interfering RNA (siRNA) RNA-mediated gene silencing. The siRNA targeting KIFC1 was designed and synthesized by Shanghai Generay Biotech Co., Ltd. (Shanghai, China), and a scrambled sequence was used as a control. The used primers for siRNA synthesis were as follows: si-KIFC1-1, sense 5′-GGUCAGUUAUGUGACCUAATT-3′, antisense 5′-UUAGGUCACAUAACUGACCTT-3′; si-KIFC1-2, sense 5′-GGUGCAAGAGCUUCAGAAATT-3,’ antisense 5′-UUUCUGAAGCUCUUGCACCTT-3′; si-KIFC1-3, sense 5′-GCUACGUAGAGAUCUACAATT-3,’ antisense 5′-UUGUAGAUCUCUACGUAGCTT-3′; si-negative control (NC), sense, 5′-AATTCTCCGAACGTGTCACGT-3′, anti-sense, 5′ACGTGACACGTTCGGAGAATT-3′. Additionally, the empty plasmids (Ov-NC) and the ETV1 overexpressed plasmids (Ov-ETV1) were established and provided by Yanzai Biotechnology (Shanghai, China).

For cell transfection, 4 × 10^4^ cells/well were seeded into a 24-well plate and maintained overnight in a cell incubator. PANC-1 cells were transferred to serum-free medium and transfected with si-NC/siRNA-KIFC1 or si-KIFC with Ov-NC/Ov-ETV1 using Lipofectamine 2000 (cat. no. 11668-027, Thermo Fisher Scientific), following the manufacturer’s instructions. After 6 h, the solution was replaced with a complete medium for routine culture. After 48 h of transfection, cells were harvested for subsequent assays.

### Cell proliferation analysis by cell counting Kit-8 (CCK-8)

Cells in the logarithmic growth phase were added to an appropriate amount of fresh complete culture medium to prepare cell suspensions and counted. The cell suspensions were seeded in 96-well plates at a density of 1 × 10^4^/well. After being maintained at 37°C and 5% CO_2_ incubator for the night, cells were transfected for 48 h. A volume of 10 μL of CCK-8 solution (cat. no. C0038, Beyotime, Shanghai, China) was added to each well. The culture plates were incubated for 2 h, and the absorbance at 480 nm was measured using a microplate reader (model: Infinite M1000 Pro, TECAN, Männedorf, Switzerland).

### Cell migration analysis by scratch assay

The 6-well plates were pre-drawn with horizontal lines (1 cm in length with at least five lines per well) on the back using a marker pen and seeded with the cells at the density of 1 × 10^6^ cells. After 48 h of transfection, the cells in each well were scratched perpendicular to the transverse line with the tip of a gun. The cell culture medium was removed and replaced with a serum-free medium for 48 h. The cells were photographed at 0 and 48 h.

### Transwell invasion assay

Cells in the logarithmic growth phase were prepared from cell suspensions and cultured in 6-well plates overnight. Cells were transfected for 12 h and digested with trypsin to prepare cell suspensions at 2 × 10^5^/mL. An 8 μm pore size Transwell chamber was coated with Matrigel (cat. no. 354234, Corning Incorporated, Corning, NY, USA). For the transwell assay, a 24-well plate was added with 500 μL medium and a transwell chamber was placed vertically into the plate. A volume of 200 μL of cell suspension was added to the chamber and cultured 48 h. The chamber was removed and washed with phosphate buffer solution. Invasive cells in the chamber were fixed with 4% paraformaldehyde and stained with crystal violet (cat. no. C0121, Beyotime). After washing and erasing the unbound crystal violet, the chamber was placed on a slide for observation and photography under a microscope (model: IX70, Olympus Corporation).

### Western blot

The cells were lysed using RIPA lysis solution (cat. no. P0013B, Beyotime) in the presence of 1mM PMSF (cat. no. ST506, Beyotime). The quality of protein samples was measured using the BCA method. The protein sample was mixed with loading buffer and put in 100°C water baths for 5–6 min. Proteins were separated by sodium dodecyl sulfate-polyacrylamide gel electrophoresis (SDS-PAGE) followed by transfer to polyvinylidene fluoride membranes (cat. no. IPVH00010, Millipore, Billerica, MA, USA). The membrane was blocked by 5% skim milk for 2 h at 37°C. Primary antibodies against E-cadherin (20874-1-AP, Proteintech), Vimentin (60330-1-Ig, Proteintech), N-cadherin (Boster, BM1573), KIFC1 (20790-1-AP, Proteintech), and GAPDH (60004-1-Ig, Proteintech) were diluted at 1:1000 and used to immerse membrane overnight at 4°C. HRP-labeled Goat Anti-rabbit IgG(H+L) secondary antibody (cat. no. 111-035-003, or cat. no. 115-035-003, Jackson ImmunoResearch) was diluted at 1:10000 and used to incubate the membrane for 2 h at 37°C. Targeted proteins were visualized using an ECL kit (P0018S; Beyotime) and photographed.

### Double luciferase reporter assay

Luciferase reporter plasmids containing wild-type or mutant KIFC1 reporters (pGL3-KIFC1-WT and pGL3-KIFC1-MUT) and the control plasmid pRL-TK were obtained from Sangon Biotech (Shanghai, China). For the luciferase reporter assay, 293T cells were seeded into 24-well plates at a density of 1 × 10^4^ cells/well and cultured overnight. Cells were replaced with serum-free medium and transfected with 0.2 μg pCDNA3.1(+)-ETV1 or pCDNA3.1(+) combined with 0.2 μg pGL3-KIF1 or 0.02 μg pRL-TK using Lipofectamine 2000 (cat. no. 11668-027, Thermo Fisher Scientific). After 6 h, the culture medium was replaced with complete medium and cultured for 48 h. Luciferase activity in the cell lysate was measured using a dual-luciferase detection system (E1910, Promega, Madison, WI, USA), according to the manufacturer’s protocol.

### Quantitative RT-RCR

The cells were harvested and lysed using 1 mL of RNAiso Plus (TRIzol, cat. no. 9109, TaKaRa, Beijing, China). Total RNA was isolated using the chloroform-isoamyl alcohol method. The RNA sample was resolved in 20 μL DEPC H_2_O. A volume of 2 μL RNA solution was used to measure RNA concentration and quality in a microplate reader (model: Infinite M1000 Pro, TECAN). A quality of 1 μg RNA was reversely transcribed using RNA PrimeScript™ RT Master Mix (RR036A, TaKaRa, Osaka, Japan). The cDNA product was amplified by real-time PCR using the Power SYBR Green PCR Master Mix (4367659, Thermo Fisher Scientific). Reaction conditions were: 50.0°C for 3 min, 95.0°C for 3 min, and 40 cycles at 95.0°C for 10 s and 60.0°C for 30 s. The following primers for the amplification were (5′-3′) KIFC1-hF, GGTGCAACGACCAAAATTACC; KIFC1-hR, GGGTCCTGTCTTCTTGGAAAC; ETV1-hF, CTGAACCCTGTAACTCCTTTCC; ETV1-hR, AGACATCTGGCGTTGGTACATA; GAPDH-hF, TGACAACTTTGGTATCGTGGAAGG; and GAPDH-hR, AGGCAGGGATGATGTTCTGGAGAG. The relative mRNA expression levels of *KIFC1*, and *ETV1* were calculated using the 2^−▵▵CT^ method with *GAPDH* as the reference gene.

### In vivo experiments

PANC-1 cells transfected with si-NC, si-KIFC1, si-KIFC1+Ov-NC, and si-KIFC1+Ov-ETV1 were harvested and their concentrations were adjusted to 3 × 10^7^ cells/mL with PBS for subsequent use.

A total of 20 SPF BALB/C male nude mice (license: SCXK[hu]2022-0004) aged 4–6 weeks were purchased from Shanghai SLAC Laboratory Animal Co., Ltd. (Shanghai, China). All the mice were maintained in the conditions of controlled temperature (24 ± 2°C) and humidity (50 ± 5%), with a 12 h light/dark cycle. During the experiment, all the mice had free access to food and water. After 7 d of acclimatization, the mice were randomly divided into four groups (n = 5 per group): si-NC, si-KIFC1, si-KIFC1+Ov-NC, and si-KIFC1+Ov-ETV1. The mice in the si-NC, si-KIFC1, si-KIFC1+Ov-NC, and si-KIFC1+Ov-ETV1 groups were respectively injected with the PANC-1 cells transfected with si-NC, si-KIFC1, si-KIFC1+Ov-NC, and si-KIFC1+Ov-ETV1 (100 μL) into the right forelimb axilla of nude mice. After the tumor cells were successfully inoculated, the mice were placed back in sterilized cages for further feeding. The long and short diameters of the tumors were measured, and tumor volume was calculated weekly. After five weeks, the mice were sacrificed by cervical dislocation, and the tumors were stripped, weighed, and photographed. Additionally, lung and liver tissues were collected from each mouse, and the tumor, lung, and liver tissues were fixed with 4% paraformaldehyde for immunohistochemistry (IHC). All procedures were performed in accordance with the National Medical Advisory Committee (NMAC) guidelines and were approved by the Institutional Animal Care and Use Committee of Southeast University (approval no. 20230925009).

### Statistical analysis

Each experiment was repeated three times, and data were expressed as mean ± standard deviation. Data analysis and graphing were performed using GraphPad Prism 5 software (GraphPad Software, San Diego, CA, USA). Statistical differences between groups and between three or more groups were analyzed using Student’s *t*-test and one-way ANOVA. Statistical significance was set at *p* < 0.05. difference.

## Results

### KIFC1 Was upregulated in pancreatic cancer and was positively correlated with TMB and tumor purity

**Figure 1:**
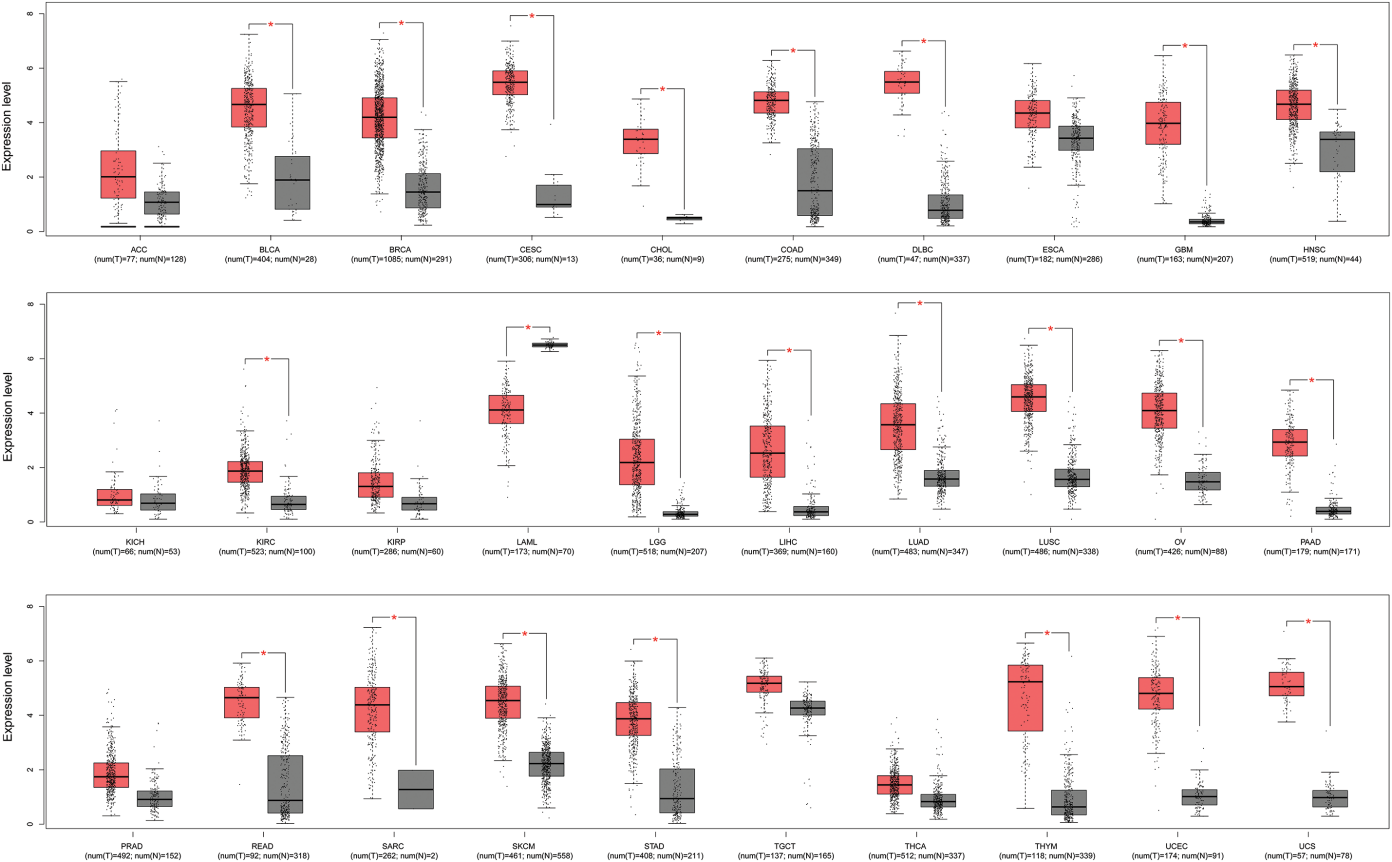
Pan-cancer analysis of KIFC1 expression in GEPIA2 database. Red box represents cancer samples and gray box represents normal control samples. * indicates *p* < 0.05. ACC, Adrenocortical carcinoma; BLCA, Bladder urothelial carcinoma; BRCA, Breast invasive carcinoma; CESC, Cervical squamous cell carcinoma and endocervical adenocarcinoma; CHOL, Cholangiocarcinoma; COAD, Colon adenocarcinoma; DLBC, Diffuse large B-cell lymphoma; ESCA, Esophageal carcinoma; GBM, Glioblastoma multiforme; HNSC, Head and neck squamous cell carcinoma; KICH, Kidney chromophobe; KIRC, Kidney renal clear cell carcinoma; KIRP, Kidney renal papillary cell carcinoma; LAML, Acute myeloid leukemia; LGG, Brain lower grade glioma; LIHC, Liver hepatocellular carcinoma; LUAD, Lung adenocarcinoma; LUSC, Lung squamous cell carcinoma; OV, Ovarian serous cystadenocarcinoma; PAAD, Pancreatic adenocarcinoma; PRAD, Prostate adenocarcinoma; READ, Rectum adenocarcinoma; SARC, Sarcoma; SKCM, Skin cutaneous melanoma; STAD, Stomach adenocarcinoma; TGCT, Testicular germ cell tumors; THCA, Thyroid carcinoma; THYM, Thymoma; UCEC, Uterine corpus endometrial carcinoma, UCS, Uterine carcinosarcoma.

**Figure 2:**
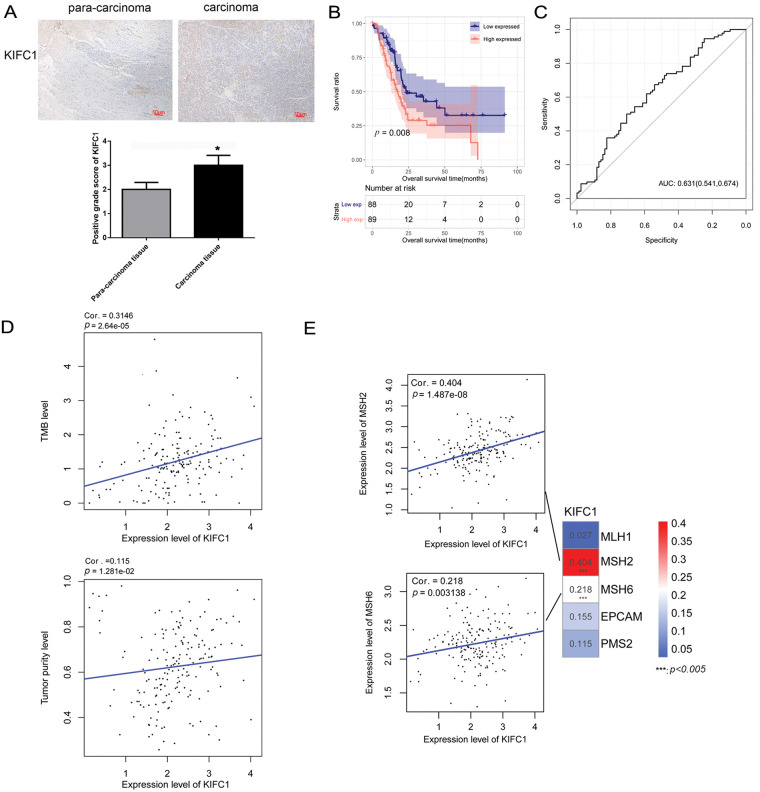
KIFC1 was upregulated in pancreatic cancer and correlated with prognosis, tumor mutation burden, and tumor purity. (A) Immunohistochemistry (IHC) detected positive KIFC1 protein expression in carcinoma tissues with para-carcinoma tissues as control. * indicates *p* < 0.05. (B) Overall survival was compared between the high KIFC1 expression population and the low KIFC1 expression population. (C) Receiver operator characteristic curve calculated the diagnostic value of KIFC1 gene expression level. The numbers in brackets represent specificity and sensitivity. (D) Scatter plot of correlation between KIFC1 gene expression level and tumor mutation burden (TMB). (E) Scatter plot of correlation between KIFC1 gene expression level and mismatch repair (MMR) genes. *** indicates *p* < 0.005.

A pan-cancer analysis of KIFC1 was performed using the GEIPA2 online database. As displayed in Fig. 1, KIFC1 was significantly upregulated in most cancers, including bladder urothelial carcinoma, breast invasive carcinoma, cervical squamous cell carcinoma and endocervical adenocarcinoma, cholangiocarcinoma, colon adenocarcinoma, diffuse large B-cell lymphoma, glioblastoma multiforme, head and neck squamous cell carcinoma, kidney renal clear cell carcinoma, brain lower grade glioma, liver hepatocellular carcinoma, lung adenocarcinoma, lung squamous cell carcinoma, ovarian serous cystadenocarcinoma, pancreatic adenocarcinoma, rectum adenocarcinoma, sarcoma, skin cutaneous melanoma, stomach adenocarcinoma, thymoma, uterine *corpus* endometrial carcinoma, and uterine carcinosarcoma (Fig. 1). The expression of KIFC1 in pancreatic cancer was further verified in our cohort samples using IHC. As shown in Fig. 2A, KIFC1 protein expression was enhanced in clinical specimens showed enhanced positive KIFC1 expression.

The samples in TCGA were further divided into high and low-KIFC1 expression groups, and the clinical information was compared between the two groups. As shown in Table 1, neoplasm histological grade was significantly different between the high KIFC1 expression group and low-KIFC1 expression group (*p* = 0.0247). Kaplan–Meier analysis suggested that high KIFC1 expression was associated with poor survival (*p* = 0.008; Fig. 2B). The area under the ROC curve was 0.631, with a specificity of 0.541 and a sensitivity of 0.674, suggesting that the expression of KIFC1 might be used as a diagnostic marker for pancreatic cancer (Fig. 2C). Univariate and multivariate Cox regression analyses demonstrated that pathological N and KIFC1 expression levels were independent prognostic factors of pancreatic cancer (Table 2).

**Table 1:** The differences of clinical information between the high KIFC1 expression group and low-KIFC1 expression group

Characteristics total cases	N of case 177	KIFC1 expression		*p*-value
		Low (N = 88)	High (N = 89)	
Age (years)				
≤65	93	51	42	1.76E−01
>65	84	37	47	
Sex				
Male	80	40	40	9.99E−01
Female	97	48	49	
Pathologic_M				
M0	79	42	37	1.97E−01
M1	5	1	4	
Pathologic_N				
N0	50	22	28	4.04E−01
N1	122	63	59	
Pathologic_T				
T1	7	4	3	2.40E−01
T2	24	16	8	
T3	141	65	76	
T4	3	2	1	
Pathologic_stage				
I	21	13	8	3.90E−01
II	145	71	74	
III	4	2	2	
IV	5	1	4	
Neoplasm histologic grade				
G1	30	22	8	2.47E−02
G2	95	45	50	
G3	48	20	28	
G4	2	1	1	
History of chronic pancreatitis				
Yes	13	4	9	2.44E−01
No	127	66	61	
History of chronic diabetes				
Yes	38	23	15	1.31E−01
No	107	48	59	

**Table 2:** The univariate and multivariate cox regression analyses of clinical characteristics

Clinical characteristics	Uni-variables cox			Multi-variables cox		
	HR	95%CI	*p*	HR	95%CI	*p*
Age (years, mean ± sd)	1.029	1.008−1.051	6.23E−03	1.109	0.997−1.041	9.00E−02
Sex (Male/Female)	0.809	0.537−1.219	3.10E−01	–	–	–
Pathologic_M (M1/M0)	0.756	0.181−3.157	7.00E−01	–	–	–
Pathologic_N (N2/N1/N0)	2.153	1.281−3.617	1.91E−03	2.118	1.222−3.669	7.45E−03
Pathologic_T (T4/T3/T2/T1)	1.556	1.003−2.413	4.77E−02	1.313	0.649−1.970	6.63E−01
Pathologic_stage (IV/III/II/I)	1.197	0.831−1.725	3.35E−01	–	–	–
Neoplasm histologic grade (G4/G3/G2/G1)	1.453	1.091−1.934	1.03E−02	1.195	0.887−1.611	2.42E−01
History of chronic pancreatitis (Yes/No)	1.177	0.562−2.465	6.65E−01	–	–	–
History of diabetes (Yes/No)	0.927	0.532−1.615	7.89E−01	–	–	–
KIFC1 expression status (High/Low)	1.745	1.151−2.647	7.94E−03	1.513	0.984−2.329	1.94E−02

Correlations between KIFC1 expression, TMB, and tumor purity were evaluated. KIFC1 expression was significantly and positively correlated with TMB and tumor purity (Fig. 2D). In addition, KIFC1 expression significantly and positively correlated with the MMR genes *MSH2* and *MSH6* (Fig. 2E).

### Correlation of KIFC1 and tumor high frequency mutated genes

**Figure 3:**
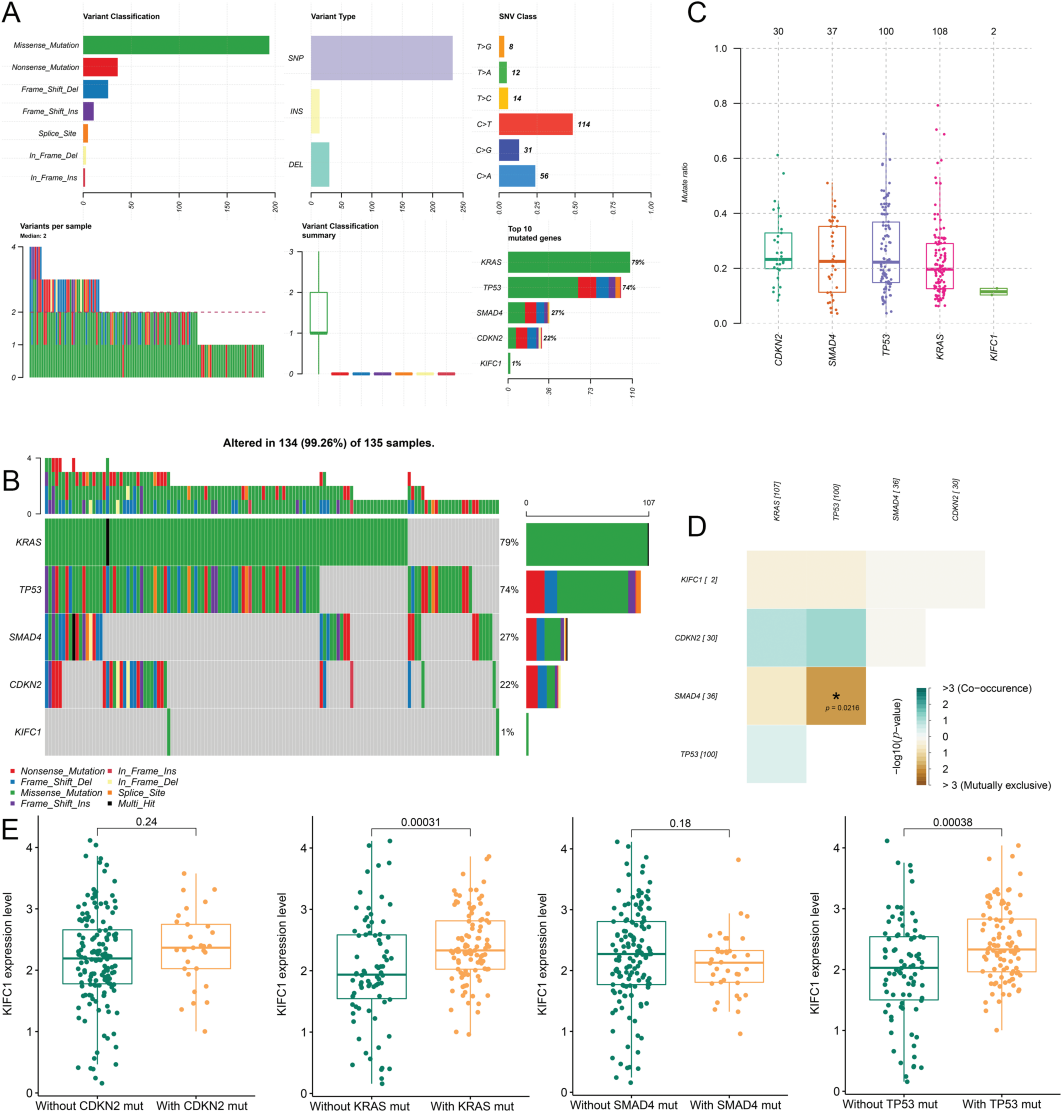
Correlation of KIFC1 and tumor high frequency mutated genes (*KRAS, TP53, CDKN2*, and *SMAD4*). (A) The mutation information of the five genes in pancreatic cancer. (B) The mutation types of the five genes in the sample. (C) The mutated rate of the five genes in the samples. (D) The co-mutation among the five genes and no co-mutation was observed between KIFC1 and KRAS/ TP53/ CDKN2/ SMAD4. (E) The correlation between the expression of KIFC1 and each tumor high frequency mutated genes (KRAS, TP53, CDKN2, and SMAD4).

Mutation information for the five genes involved in pancreatic cancer is shown in Fig. 3A. *KRAS* mutations (79%) occurred in most tumor samples, followed by *TP53* mutations (74%), *SMAD4* mutations (27%), and *CDKN2* mutations (22%) (Fig. 3B). Additionally, the mutation of *KIFC1* was found in the two samples, both of which were missense mutations (Fig. 3B). Furthermore, *KRAS*, *TP53*, *CDKN2*, and *SMAD4* were all high-frequency mutated genes with high mutation rates (Fig. 3C). No co-mutations were observed between *KIFC1* and *KRAS*/ *TP53*/ *CDKN2*/ *SMAD4* (Fig. 3D). Subsequently, according for mutations in *KRAS*, *TP53*, *CDKN2*, and *SMAD4*, the correlation between *KIFC1* and each gene was investigated. The expression of *KIFC1* had significant correlation with *KRAS* mutation (*p* = 0.00031) and *TP53* mutation (*p* = 0.00038) (Fig. 3E).

### GSEA analysis identified KIFC1 associated pathways

**Figure 4:**
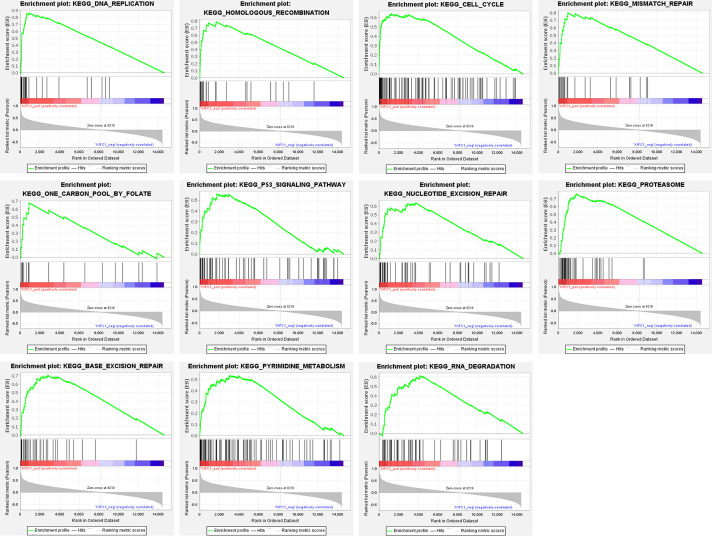
GSEA EN-plot of KEGG pathways with significant correlation of KIFC1 expression level.

GSEA analysis identified 11 KEGG pathways that were significantly correlated with KIFC1 expression, including “DNA replication” (NES = 2.11, FDR = 0.011), “homologous recombination” (NES = 1.99, FDR = 0.031), “cell cycle” (NES = 1.99, FDR = 0.021), “mismatch repair” (NES = 1.98, FDR = 0.022), “one carbon pool by folate” (NES = 1.92, FDR = 0.038), “p53 signaling pathway” (NES = 1.91, FDR = 0.038), “nucleotide excision repair” (NES = 1.86, FDR = 0.046), “proteasome” (NES = 1.85, FDR = 0.045), “base excision repair” (NES = 1.83, FDR = 0.046), “pyrimidine metabolism” (NES = 1.81, FDR = 0.047), and “RNA degradation” (NES = 1.79, FDR = 0.047) (Fig. 4).

### Knockdown of KIFC1 Suppressed Proliferation, Migration, and Invasion of Pancreatic Cancer Cells

**Figure 5:**
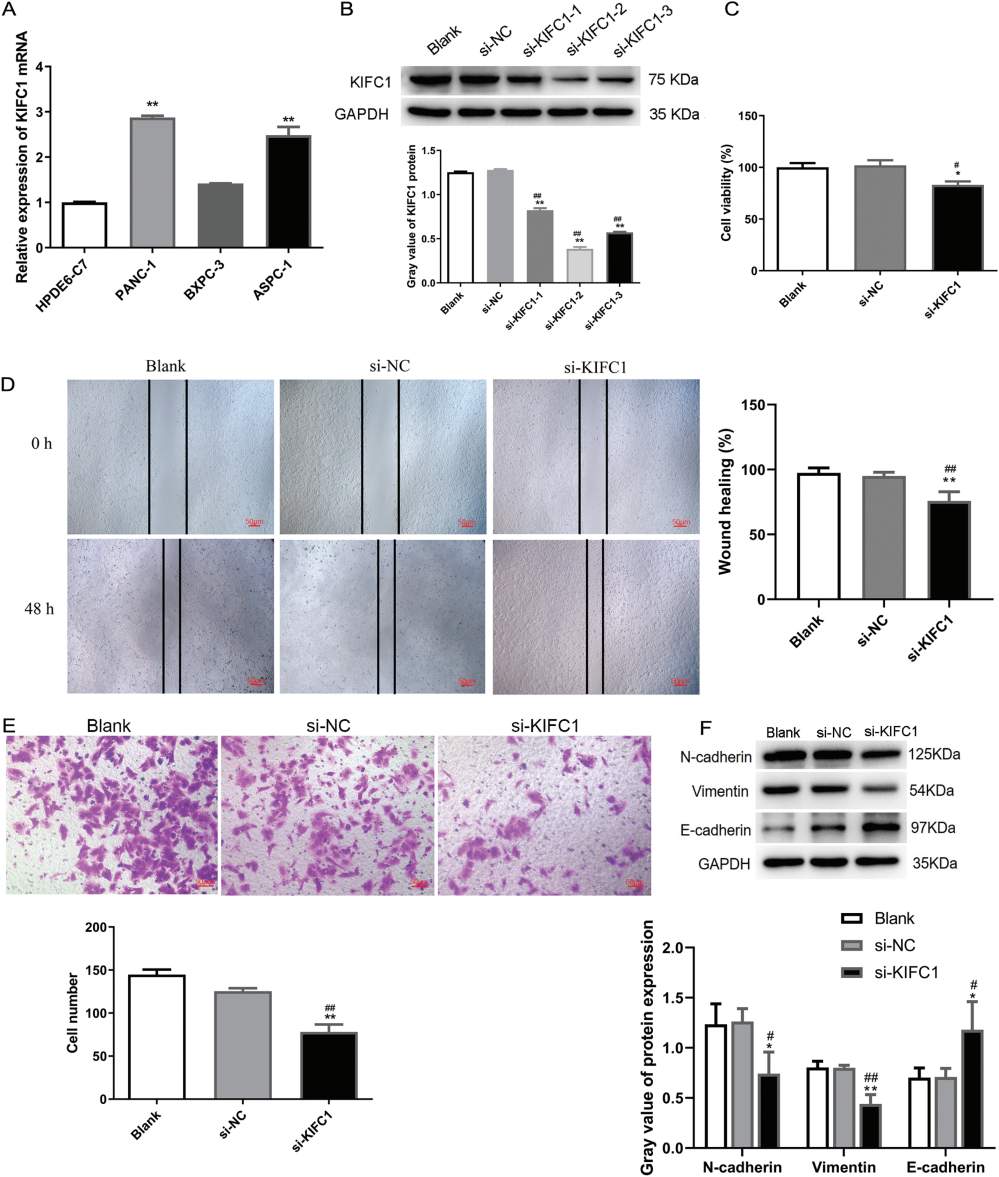
Knockdown of KIFC1 suppressed proliferation, migration, and invasion of pancreatic cancer cells. (A) qPCR detected KIFC1 mRNA level in three pancreatic cancer cell lines with HPDE6-C7 as control. (B) KIFC1 protein expression in PANC-1 cells transduced by three siRNAs targeted to KIFC1 (si-KIFC-1, -2, and -3) and siRNA negative control (si-NC) was determined by western blot and quantified by gray analysis. GAPDH served as internal control. (C) Cell proliferation was assessed by CCK-8 assay. (D) Cell migration was evaluated by scratch test. (E) Invasive cells were determined by transwell assay. (F) EMT-associated protein expression was determined by western blot and quantified by gray analysis. * and ** indicate *p* < 0.05 and *p* < 0.01 compared with blank. # and ## indicate *p* < 0.05 and *p* < 0.01 compared with si-NC.

To further clarify the KIFC1 expression pattern, we determined its mRNA levels in the three pancreatic cancer cell lines. As shown in Fig. 5A, KIFC1 mRNA was significantly increased in pancreatic cancer PANC-1 and ASPC-1 cells, however, not in pancreatic cancer BXPC-3 cells, compared to the control HPDE6-C7 cells. The cell line PANC-1 with the highest KIFC1 expression was selected for subsequent experiments.

To explore the function of KIFC1 in pancreatic cancer cells, KIFC1 was knocked down in PANC-1 cells by transfection with the three siRNAs (Fig. 5B). The si-KIFC1-2, which showed the strongest interference effect, was selected for subsequent cell experiments. Experiments on cell properties revealed that cell proliferation, migration, and invasion in KIFC1 knocked-down cells were attenuated compared with control si-NC-transduced cells (Fig. 5C–E). In addition, the knockdown of KIFC1 inhibited N-cadherin and Vimentin expression and promoted E-cadherin expression (Fig. 5F), indicating that KIFC1 knockdown suppressed the EMT capacity of pancreatic cancer cells.

### ETV1 targeted and increased KIFC1 expression

**Figure 6:**
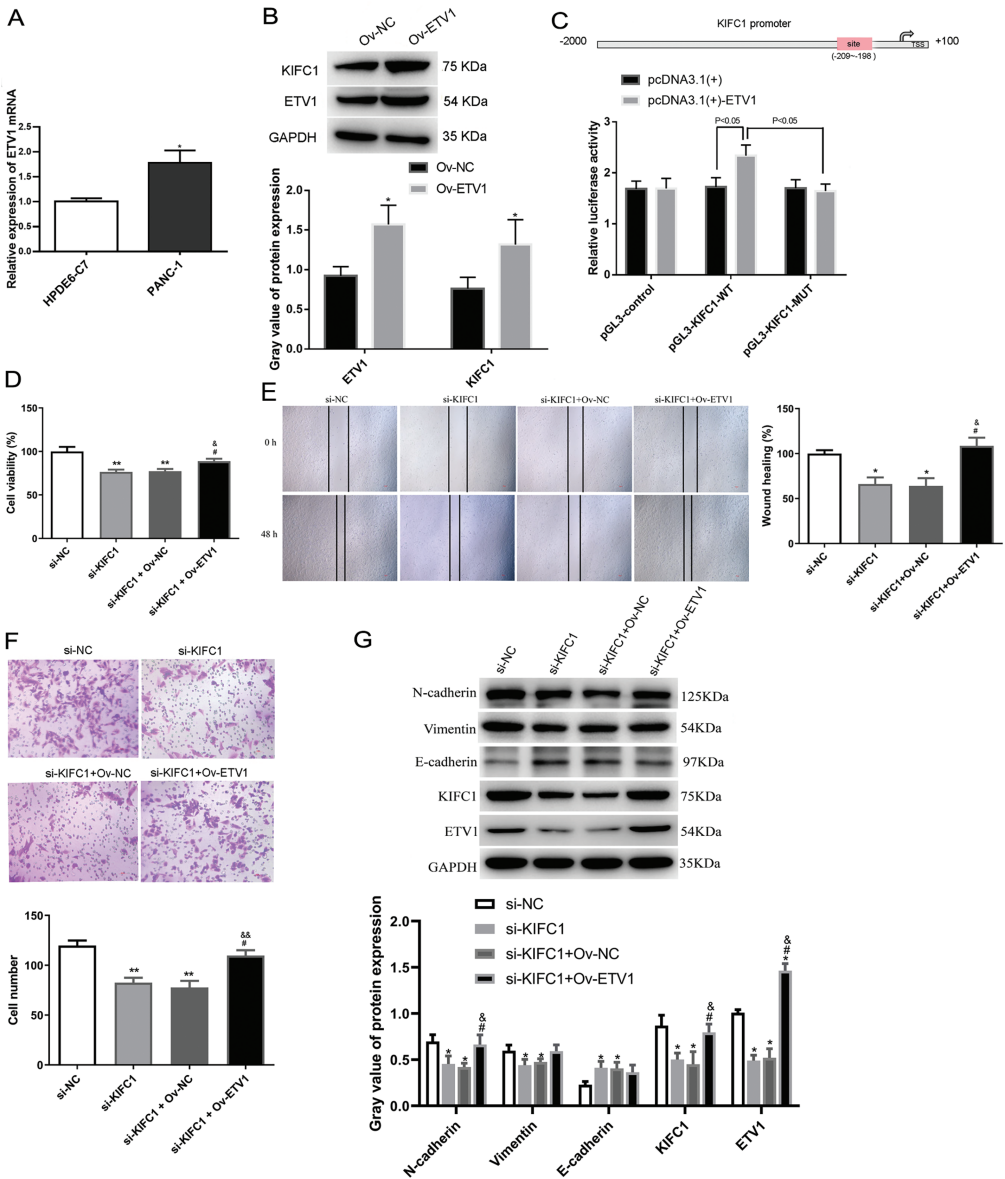
ETV1 overexpression reversed the inhibiting function of KIFC1 knockdown on proliferation, invasion and EMT *in vitro*. (A) ETV1 mRNA level was measured in PANC-1 cells with HPDE6-C7 as a control. (B) ETV1 and KIFC1 protein expressions were determined in PANC-1 cells overexpressed ETV1 by western blot with vector as negative control (Ov-NC). (C) Luciferase gene reporter assay was performed to investigate the relationship between ETV1 and KIFC1. (D–F) Cell proliferation (D), migration (E), and invasion (F) were determined. (G) EMT-associated protein expression was determined in PANC-1 cells with KIFC1 knockdown and ETV1 overexpression. * and ** indicate *p* < 0.05 and *p* < 0.01 compared with si-NC or Ov-Nc. # indicates *p* < 0.05 compared with si-KIFC1. & and && indicate *p* < 0.05 and *p* < 0.01 compared with si-KIFC1+Ov-NC.

ETV1 mRNA levels were higher in PANC-1 cells than those in control HPDE6-C7 cells (Fig. 6A). To determine the regulatory function of ETV1 in KIFC1 expression, we measured KIFC1 expression level in ETV1-overexpressed PANC-1 cells. Fig. 6B shows that the KIFC1 protein expression level was significantly increased by ETV1 overexpression compared to that in the negative control. As the analysis in the hTFtarget data, (http://bioinfo.life.hust.edu.cn/hTFtarget#!/prediction) (accessed on 5 March 2025), ETV1 had the potential binding site on the KIFC1 promoter at −209~−198 sites (Fig. 6C). Using a luciferase gene reporter assay, we observed that ETV1 overexpression increased the luciferase activity of the wild-type KIFC1 reporter, however, had no effect on the luciferase activity of the mutant KIFC1 reporter (Fig. 6C), indicating that ETV1 regulates KIFC1 expression by targeting KIFC1 transcription.

### ETV1 overexpression reversed the anti-proliferation and -invasion function of KIFC1 knockdown In vitro

Next, we explored the functional interaction between ETV1 and KIFC1 in affecting cell proliferation, migration, and invasion in PANC-1 cells overexpressing ETV1 and knocking down KIFC1. As shown in Fig. 6D, knockdown of KIFC1 alone reduced cell viability, whereas KIFC1 knockdown and ETV1 overexpression cotreatment restored cell viability, suggesting that ETV1 overexpression reversed the anti-proliferative function of KIFC1 knockdown. The anti-migratory and anti-proliferative effects of KIFC1 knockdown were also reversed by cotreatment with ETV1 overexpression and KIFC1 knockdown (Fig. 6E,F).

### ETV1 overexpression reversed the inhibiting effect of KIFC1 knockdown on EMT-associated protein expression in vitro

EMT-associated protein expression was determined in PANC-1 cells with KIFC1 knockdown and ETV1 overexpression. ETV1 overexpression still aroused the increased KIFC1 protein expression even after KIFC1 knockdown (Fig. 6G). The expression of ETV1 was significantly downregulated in cells transfected with si-KIFC1 and si-KIFC1+Ov-NC compared with that in si-NC cells (*p* < 0.05); as well as cotreatment of KIFC1 knockdown and ETV1 overexpression further up-regulated ETV1 expression (*p* < 0.05, Fig. 6G). Among the EMT-associated proteins, decreased N-cadherin and vimentin expression and increased E-cadherin expression by KIFC1 knockdown were all reversed by ETV1 overexpression; however, only N-cadherin showed a significant change (Fig. 6G).

### ETV1 overexpression reversed the action of KIFC1 knockdown in vivo

**Figure 7:**
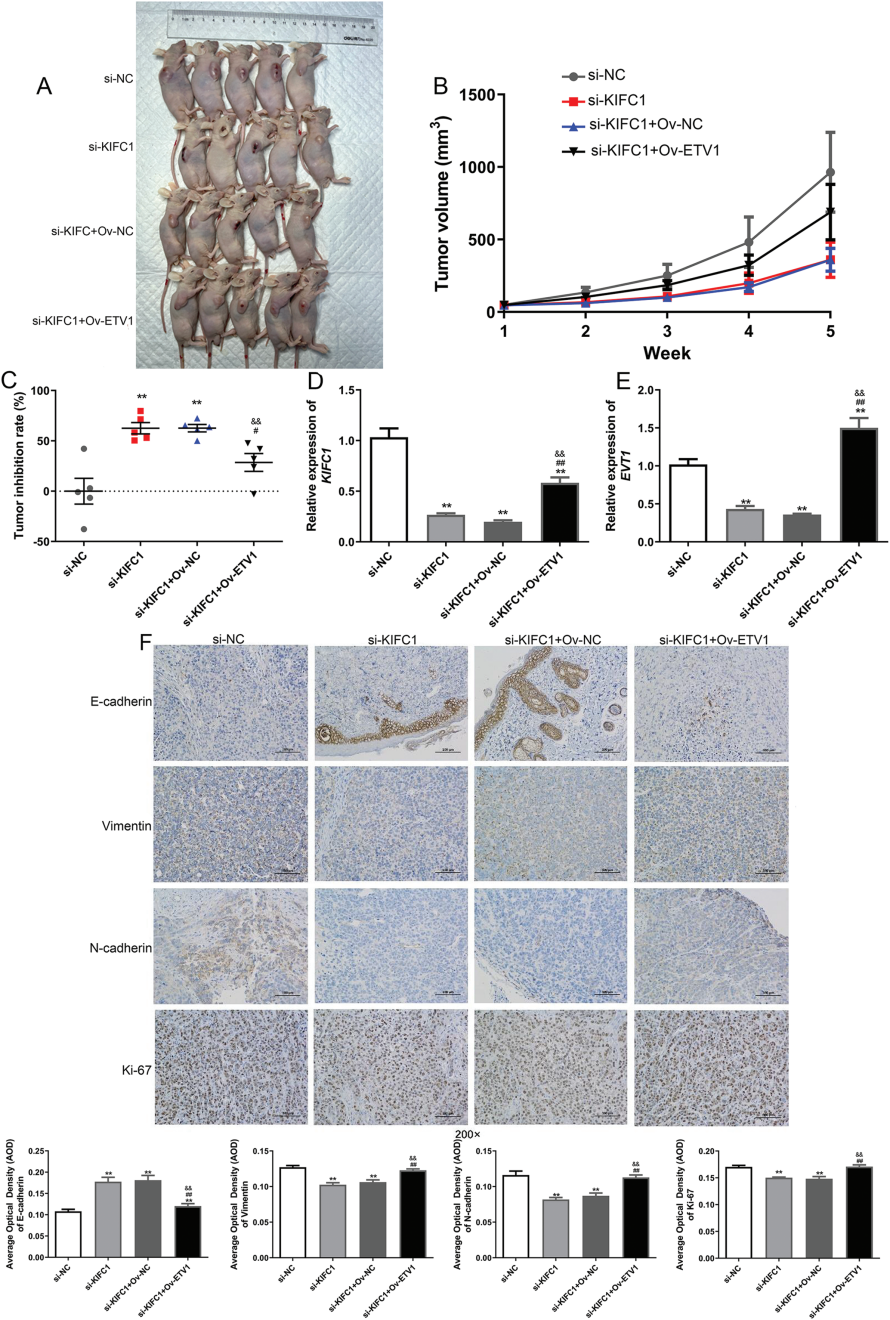
ETV1 overexpression reversed the inhibitory action of KIFC1 knockdown in tumor growth and EMT *in vivo*. (A) The size of tumor in different groups at week 5. (B) The changes of tumor volume with the increase of administration time. (C) The tumor inhibition rate in the different groups at week 5. (D) The *KIFC1* mRNA expression level in the different groups at week 5. (E) The *ETV1* mRNA expression level in the different groups at week 5. (F) The expression of EMT-associated proteins in the different groups detected using immunohistochemistry. ** indicate *p* < 0.01 compared with si-NC. # and ## indicate *p* < 0.05 and *p* < 0.01 compared with si-KIFC1. && indicate and *p* < 0.01 compared with si-KIFC1+Ov-NC.

We also investigated the interaction between ETV1 and KIFC1 *in vivo*. As shown in Fig. 7A, the tumor size after KIFC knockdown was lower than that in the si-NC group, whereas ETV1 overexpression increased the tumor size induced by si-KIFC. With increasing feeding time, the tumor volumes in the different groups gradually increased (Fig. 7B). However, at week 5, the tumor volumes in the si-KIFC and si-KIFC+Ov-NC groups were the lowest (Fig. 7B). The tumor inhibition rate was analyzed and the tumor inhibition rates in the si-KIFC and si-KIFC+Ov-NC groups were significantly higher than those in the si-NC mice (*p* < 0.05), whereas ETV1 overexpression reduced the tumor inhibition rates induced by si-KIFC1 (*p* < 0.05, Fig. 7C). These results indicate that KIFC1 knockdown could inhibit tumor growth in pancreatic cancer, but ETV1 expression could reverse the action of KIFC1.

Furthermore, the expression levels of KIFC1 and ETV1 in the different groups were determined. Compared to the si-NC group, KIFC1 and ETV1 expression was significantly downregulated in the si-KIFC and si-KIFC+Ov-NC groups (*p* < 0.05), whereas ETV1 overexpression evidently up-regulated the expression caused by si-KIFC1 (*p* < 0.05, Fig. 7D,E). Next, the expression of EMT-associated proteins (E-cadherin, N-cadherin, Vimentin, and Ki-67) in different tomato tissues was detected using IHC. The E-cadherin expression was up-regulated after KIFC1 knowkdown, whereas the expression of N-cadherin, Vimentin, and Ki-67 were down-regulated compared with that in the si-NC group (Fig. 7F). However, in the si-KIFC1+Ov-ETV1 group, E-cadherin expression was significantly reduced (*p* < 0.05), whereas the expression of N-cadherin, Vimentin, and Ki-67 was evidently enhanced compared with that in the si-KIFC1 group (*p* < 0.05, Fig. 7F). These results suggest that KIFC1 knockdown suppresses EMT progression in pancreatic cancer; however, ETV1 overexpression partially reverses the inhibitory effect of KIFC1 knockdown on EMT.

## Discussion

Pancreatic cancer is the leading cause of cancer-related deaths worldwide and its global burden has more than doubled over the last 25 years [19]. Previous studies have employed a series of bioinformatic analyses to establish an immune-associated programmed cell death model for lung adenocarcinoma (LUAD) [20] and identified nine T-cell exhaustion-related genes for the prediction of LUAD prognosis [21], which implied the significance of bioinformatics analysis. KIFC1 expression is closely associated with pancreatic cancer [9,22]; however, the underlying mechanisms remain unclear. Therefore, the current study first used bioinformatic analyses to observe that KIFC1 expression was increased in pancreatic cancer and positively correlated with tumor mutation burden, tumor purity, mismatch repair, and KRAS and TP53 mutation. High expression of KIFC1 was significantly associated with a poor prognosis of pancreatic cancer. Knockdown of KIFC1 suppresses the proliferation, migration, and invasion of pancreatic cancer cells and tumor growth *in vivo*. ETV1 overexpression increased KIFC1 expression and affected KIFC1 transcription. ETV1 overexpression reversed the role of KIFC1 knockdown in inhibiting the proliferation, invasion, migration, and EMT of pancreatic cancer cells and tumor growth *in vivo*. The ETV1/KIFC1 axis was involved in the abnormal expression of EMT-associated proteins and induced the proliferation, invasion, and migration of pancreatic cancer cells, thereby contributing to the progression of pancreatic cancer.

KIFC1 is an important kinesin for cancer cells and allows for division and survival [23,24]. As in the reported studies, KIFC1 is widely expressed in cancer cells, such as prostate, ovarian, breast, lung, and endometrial cancers [25], and we report here, pancreatic cancer. A hallmark of pancreatic cancer is the prevalence of cancerous mutations in the KRAS oncogene, which plays a key role in the development and maintenance of pancreatic tumors [26]. Furthermore, KRAS-TP53 genomic co-alterations are associated with an immune rejection microenvironment, chemotherapy resistance, and poor survival in patients with pancreatic ductal adenocarcinoma [27]. Our study showed that KIFC1 expression was significantly correlated with KRAS and TP53 mutations, further suggesting the importance of KIFC1 in pancreatic cancer.

Elevated KIFC1 levels are important for tumorigenesis and tumor progression. Cancer cell properties affected by KIFC1 include aerobic glycolysis [28], proliferation and invasion [25], drug resistance [29], and EMT [30]. Consistent with the aforementioned results, our data showed that the knockdown of KIFC1 restricted the proliferation, invasion, migration, and EMT of pancreatic cancer cells and suppressed tumor growth *in vivo*. E-cadherin, Vimentn, and N-cadherin belong to EMT-associated markers. EMT is a process responsible for epithelial cell transformation into mesenchymal and epithelial characteristics, and is indispensable for tumor metastatic progression by enhancing migration and invasion properties [31]. Combined with uncontrolled proliferation, invasion, and migration through the extracellular matrix are necessary steps in cancer progression [32]. In a review by Sun et al., high levels of KIFC1 were closely correlated with positive lymph node metastasis and advanced tumor node metastasis [33]. KIFC1 overexpression is associated with poor clinicopathological characteristics in various malignant tumors [33]. Based on these results, it can be concluded that KIFC1 is essential for the development and progression of pancreatic cancer, and may be a diagnostic and therapeutic target.

Although it is widely believed that KIFC1 is highly expressed in cancer cells, how KIFC1 gene expression is elevated remains largely unknown. The major strength of the present study is that KIFC1 promoter transcription and expression were targeted by the transcription factor ETV1. Studies have reported that KIFC1 expression can be targeted by miRNAs through post-transcriptional regulation [10,34] or by transcription factors E2F and TCF-4 through transcriptional regulation [8,23]. This is the first study to identify ETV1 as a transcriptional regulator of KIFC1 in pancreatic cancer cells. ETV1 is active in various cancers and plays a crucial role in cancer progression by affecting cell proliferation and metastasis [35,36]. Clinical evidence supports ETV1 as a prognostic factor in patients with cancer [37,38]. To date, few studies have described the role of ETV1 in pancreatic cancer. According to a previous report, ETV1 is increased and essential for the metastatic progression of pancreatic cancer [15]. Our data showed that ETV1 overexpression increased KIFC1 expression and reversed the anti-proliferative and anti-invasive effects of KIFC1 knockdown. Collectively, it can be concluded that the ETV1/KIFC axis is crucial for the development and progression of pancreatic cancer by affecting cancer cell properties, such as proliferation, invasion, and migration.

However, there are some limitations in our study. The upstream and downstream relationship of KIFC1 and ETV1 need to be further confirmed by other technologies. Additionally, the KIFC1/ETV1 as novel targets to treat pancreatic cancer should be explore in clinical practice.

## Conclusions

The present study clarified the tumor-promoting action of the KIFC1/ETV1 axis in pancreatic cancer by strengthening cell proliferation, migration, and invasion. Our findings may be useful for understanding biological mechanisms and identifying new therapeutic targets for pancreatic cancer.

## Data Availability

All data generated or analysed during this study are included in this article.
